# Do semantic contextual cues facilitate transfer learning from video in toddlers?

**DOI:** 10.3389/fpsyg.2015.00561

**Published:** 2015-05-12

**Authors:** Laura Zimmermann, Alecia Moser, Amanda Grenell, Kelly Dickerson, Qianwen Yao, Peter Gerhardstein, Rachel Barr

**Affiliations:** ^1^Department of Psychology, Georgetown UniversityWashington, DC, USA; ^2^Department of Psychology, Binghamton UniversityBinghamton, NY, USA; ^3^Institute of Child Development, University of Minnesota, MinneapolisMN, USA; ^4^Army Research Laboratory, Human Research and Engineering Directorate, Aberdeen Proving GroundAberdeen, MD, USA

**Keywords:** transfer deficit, context learning, imitation, social learning, learning from screen media, memory binding

## Abstract

Young children typically demonstrate a *transfer deficit*, learning less from video than live presentations. Semantically meaningful context has been demonstrated to enhance learning in young children. We examined the effect of a semantically meaningful context on toddlers’ imitation performance. Two- and 2.5-year-olds participated in a puzzle imitation task to examine learning from either a live or televised model. The model demonstrated how to assemble a three-piece puzzle to make a fish or a boat, with the puzzle demonstration occurring against a semantically meaningful background context (ocean) or a yellow background (no context). Participants in the video condition performed significantly worse than participants in the live condition, demonstrating the typical *transfer deficit effect*. While the context helped improve overall levels of imitation, especially for the boat puzzle, only individual differences in the ability to self-generate a stimulus label were associated with a reduction in the transfer deficit.

## Introduction

Infants and young children perform more poorly on tasks involving transfer of learning from television to real-life situations than in direct face-to-face interactions. This finding, which has been termed the *transfer deficit* (Barr, 2010, 2013), is supported by data from multiple investigations including imitation (Barr and Hayne, 1999; Flynn and Whiten, 2008; Nielsen et al., 2008; Zack et al., 2009; Simcock et al., 2011; Dickerson et al., 2013), object retrieval (Troseth and DeLoache, 1998; Troseth et al., 2006), self-recognition (Suddendorf et al., 2007), and object recognition tasks (Carver et al., 2006; Simcock and Dooley, 2007). For example, Dickerson et al. (2013) reported a transfer deficit that persisted across early childhood. Using an imitation procedure, they modeled assembly of a 3-piece puzzle of a fish or a boat via either a live or televised demonstration. Toddlers (2- and 2.5-year-olds) imitated significantly fewer gestures and goals following a video demonstration than a live demonstration. This transfer deficit is problematic for early childhood learning, especially with the increased popularity of computers, television, and other interactive media as teaching tools for infants and toddlers (Rideout, 2013, 2014).

One account of the deficit notes that it may be challenging for children to perceptually match features between encoding and retrieval when the features undergo changes in color, brightness, motion, and depth information between the demonstration (e.g., video) and the test. These changes increase the *transfer distance* (Barnett and Ceci, 2002) between the training and test situations; that is, the degree of similarity between encoding and retrieval of new information. Other accounts focus on the effect that transfer distance has on specific aspects of memory processing including symbolic understanding (Troseth and DeLoache, 1998), memory flexibility (Hayne, 2004) and memory binding (Olson and Newcombe, 2014). The transfer deficit can be ameliorated by manipulations that reduce transfer distance and increase memory flexibility (see Barr, 2010, 2013; Troseth, 2010 for review and discussion) through repetition (Barr et al., 2007), social engagement (Tennie et al., 2006; Nielsen et al., 2008; Subiaul et al., 2012), contingency cues (eye contact, directed gaze, directed pointing; Csibra and Gergely, 2006), and increased perceptual realism (Simcock and DeLoache, 2006; Simcock et al., 2011). Given the well-established beneficial role of context in learning, the present study sought to address whether the inclusion of a semantically meaningful context would ameliorate the transfer deficit on an established imitation task in 2- and 2.5-year-olds.

### Effect of Context

Context is the physical, temporal, and affective or internal environment within which an event occurs (Bouton, 1993). The role of context in learning is well established in both the animal and human learning literature (Bouton, 1993; Boller et al., 1996; see also Rescorla and Wagner, 1972; Barnat et al., 1996; Learmonth et al., 2004). Bouton (1993) found that consistency between the context present at training (encoding) and the context present at test facilitates memory retrieval. The benefit of contextual consistency has also been found in studies of infants using the mobile conjugate reinforcement procedure (e.g., Borovsky and Rovee-Collier, 1990) and a deferred imitation paradigm (Hayne et al., 2000). While the context and cues are often discussed experimentally as separate and relatively independent entities, early in development context and cues are thought to be parts of a single encoded event (Spear and McKinzie, 1994). When these parts are congruent, object recognition should be more precise (Oliva and Torralba, 2007). Thus, children may be better able to identify a fish in the ocean than on a mountaintop, or against a solid (non-specific) background.

An encoded event is generally seen as the result of memory binding. Memory binding is the process of encoding the relations among stimuli that co-occur spatially or temporally (Cohen and Eichenbaum, 1993). This process is critical to the ability to integrate visual background context into a memory for central foreground object details. There is a long developmental trajectory of memory binding across childhood that has been linked to hippocampal development (Raj and Bell, 2010; Olson and Newcombe, 2014), but investigation across early childhood has not been systematic due to differences in approaches and measurement across age.

Early in development, cue information appears to be inextricably bound to other memory attributes, including attributes of the context in which the event occurs, such as the background scene, making transfer of learning outside a particular context challenging (Spear and McKinzie, 1994). Boller et al. (1996) reported that infants’ memory retrieval was robust in the presence of the training context, but degraded when the context was changed or removed. Additional work has demonstrated that memory retrieval of 6-month-olds is highly context-specific, such that a contextual change (i.e., original mobile cue in a novel context) disrupts retention following as little as a 24-h delay (Borovsky and Rovee-Collier, 1990; Hartshorn and Rovee-Collier, 1997). Development then is characterized by a decrease in contextualized learning and a subsequent increase in flexibility of memories to withstand changes in context.

Hayne et al. (2000) found similar effects with 6-month-olds using a deferred imitation paradigm. They found that when the context changed between demonstration and test (e.g., from home to laboratory), 6-month-old infants were no longer able to imitate the target actions, while older children, 12- and 18-month-olds, were successful at transferring learning across a context change from the home to the laboratory setting. These studies suggest that learning is highly context-specific early in infancy, and that context features might bind with other memory attributes to form a single cue representation. Hayne (2004) noted that this high degree of memory specificity constrains memory flexibility and generalization of learning to new settings, and argued that the ability to use memory more flexibly develops across infancy and childhood.

Memory binding has also been examined using visual recognition memory paradigms during infancy and has revealed evidence of fragile memory binding (Richmond and Nelson, 2009). Using precise eye-tracking techniques, Richmond and Nelson (2009) demonstrated that infants could encode memories based on relationships between images. However, with age-dependent experience, children learn to disregard or de-emphasize less relevant contextual information and focus more on central cues (Bornstein et al., 2011). An increase in hippocampal volume in infancy may help explain the rapid changes in memory binding (Olson and Newcombe, 2014). By age 2, there is a shift in spatial coding and representation as children are able to encode multiple spatial locations and maintain them across a delay (Sluzenski et al., 2004).

Less work investigating the memory binding capacities of toddlers is available. Studies with 4- to 6-year-olds have used protocols adapted from studies of adults. In particular, children display difficulty with tasks that involve reporting the combination of visual foreground object and contextual background cues, suggesting that memory binding continues to develop into the preschool years (Sluzenski et al., 2006; Lloyd et al., 2009). Specifically, there were age-related increases in performance between 4 and 6 years (Lloyd et al., 2009). There were also age-related differences between children and adults; 4- to 6-year-old children performed significantly worse on the combined condition than adults (Sluzenski et al., 2006). One possible explanation for the poor performance of 4- to 6-year-olds in these studies was memory binding or retrieval deficits, but another possibility is that the task (verbal report) was too taxing for this age range. A non-verbal measure is likely to provide a better index of memory binding in younger children.

More recently, Newcombe et al. (2014) used an episodic memory search paradigm to examine memory binding in young children, and found systematic age-related increases in search performance among 15- to 72-month-olds. Older children remembered more items (toys) across different rooms (contexts). Younger children (under 26 months) remembered more locations when they were given a label than when they were not, but older children (34- to 56-month-olds) did not benefit from a label cue. Other studies have demonstrated that the scale of the contextual cue also critically determines whether toddlers will use the cue effectively or not. For example, Deloache et al. (2004) demonstrated that transfer from a small-scale model to a larger-scale model was significantly easier than transfer from a small-scale model to a real room and vice versa for 2.5- and 3-year-olds. In the present study, we therefore reduced the demands on toddlers by testing them with fewer items and by testing the effect of contextual cues on transfer within a much smaller space.

Taken together, the studies discussed above demonstrate age-related changes in memory binding and processing of contextual cues from infancy to school-age. These changes are associated with a host of developmental changes in memory processing. Older children have better memory capabilities; that is, they encode more efficiently and are better able to equate and integrate information across different contexts compared to younger children, showing better memory flexibility across time (Hayne, 2004; Barr, 2013). Contextual cues may be weighted and bound differently as a function of age and complexity (Deloache et al., 2004; Olson and Newcombe, 2014). Infants may encode background contextual information at the expense of central information, resulting in disruption in memory processing when the context changes (e.g., Borovsky and Rovee-Collier, 1990; Shields and Rovee-Collier, 1992; Boller et al., 1996; Hayne et al., 2000). Toddlers may progress from fused memory representations that have both central and background information, to memory representations that contain primarily central cue information, resulting in neither a disruptive nor a facilitative effect of context. Later in development, they may progress to more flexible adult-like memory that has both background and central information stored in a relational network that can be accessed depending upon the specific situation (Sluzenski et al., 2006; Lloyd et al., 2009; Olson and Newcombe, 2014).

### Effect of Visually Meaningful Cues on Memory

Manipulations of context in the research discussed above were highly distinctive (large changes to brightly colored crib liners, different physical locations), but not *iconic*. An iconic – or semantically related – visual context is thought to tap into the rich background knowledge and the extensive visual experience of the observer, and thus facilitate performance (Simcock and DeLoache, 2006; Pereira and Smith, 2009). An example would be depicting a car on a street, as compared to an arbitrary context (Biederman, 1972; Oliva and Torralba, 2007). A related visual context can direct spatial attention to important features in a display and facilitate adult memory for a visual context (Chun and Jiang, 1998). This is especially relevant during early childhood as visual context may be a semantically meaningful cue for young children, who have a smaller verbal semantic network available to them. A semantically meaningful context has been shown to facilitate recognition and object search in 2-year-olds (Pereira and Smith, 2009). Finally, 24-month-olds perform significantly more target actions from a picture book when drawings are iconic photographs than when they are line drawings (Simcock and DeLoache, 2006). The potential advantage conveyed by related context may be especially relevant during early childhood, as contextual cues may increase the probability of retrieving a semantically meaningful target. Young children have a smaller verbal semantic network available to them, and thus the presence of an iconic visual context may produce a greater level of performance increase by providing more cues with which to access the memory. The role that visual context – specifically semantically meaningful scenes – plays on learning and memory in toddlers is explored here.

### The Present Study

The present study adopted methodology from Dickerson et al. (2013), using the same 3-piece boat and fish puzzle apparatus. The primary research question was whether the presence of a semantically meaningful visual context would ameliorate the transfer deficit on the puzzle imitation task. The reproduction of demonstrated gestures and final goal state of the puzzle (fish or boat) during the task, were coded in the present study. Groups of 2- and 2.5-year-old children were tested on the puzzle imitation task following a live or video demonstration. These ages were selected because the Dickerson et al. (2013) test demonstrated that performance in this age range is neither at floor nor at ceiling for the puzzle task. Half of the children were assigned to a meaningful semantic context condition and the other half were not. Performance was compared to baseline controls that never saw a demonstration. The current study extends previous work by manipulating the presence of a semantically meaningful context to examine whether increasing semantic congruence can ameliorate the transfer deficit.

We sought to link context to confines of a smaller space than previous memory binding studies in large rooms (Newcombe et al., 2014) using a task that 2- to 3-year-olds has been successful on. Additionally, we intended to extend previous work on the transfer deficit to include the role of context. Consistent with the memory binding accounts of context, we hypothesized that the presence of a visual semantic context (i.e., ocean) would facilitate imitation of a demonstrated goal and gestures. Given previously documented age-related changes in imitation on this task (Dickerson et al., 2013), we hypothesized that older children would be more successful in transfer tasks compared to younger children. Furthermore, applying the transfer deficit concept (Barr, 2010, 2013) to this design, we predicted that the addition of a semantically meaningful visual context would ameliorate the transfer deficit.

## Materials and Methods

### Participants

The study included 165 typically developing children (87 boys) from two metropolitan areas. Independent groups of children were tested at 2 years (*N* = 88, *M* age = 24 months 16 days, SD = 11.46 days, range 23–25 months) and 2.5 years (*N* = *77*, *M* age = 30 months 16 days, SD = 25 days, range 28–31 months). Participants were primarily Caucasian (79.9%) and from college-educated families (*M* years of education = 17.26, SD *=* 1.26). The remaining 20% of the sample included the following races: Mixed (14.6%), African–American (1.8%), Asian (0.9%), and not reported (2.8%). Additionally, 6.5% of the sample was Latino. The mean rank of socioeconomic index (SEI; Nakao and Treas, 1994) was 74.14 (SD = 19.12) based on 127 families (76%). Additional children were excluded from the analysis for the following reasons: eight due to experimenter error, three for technical error, five for failure to interact with the experimental stimuli, 14 due to parental interference, and 20 for interacting with the stimuli prior to test.

### Apparatus

**FIGURE 1:**
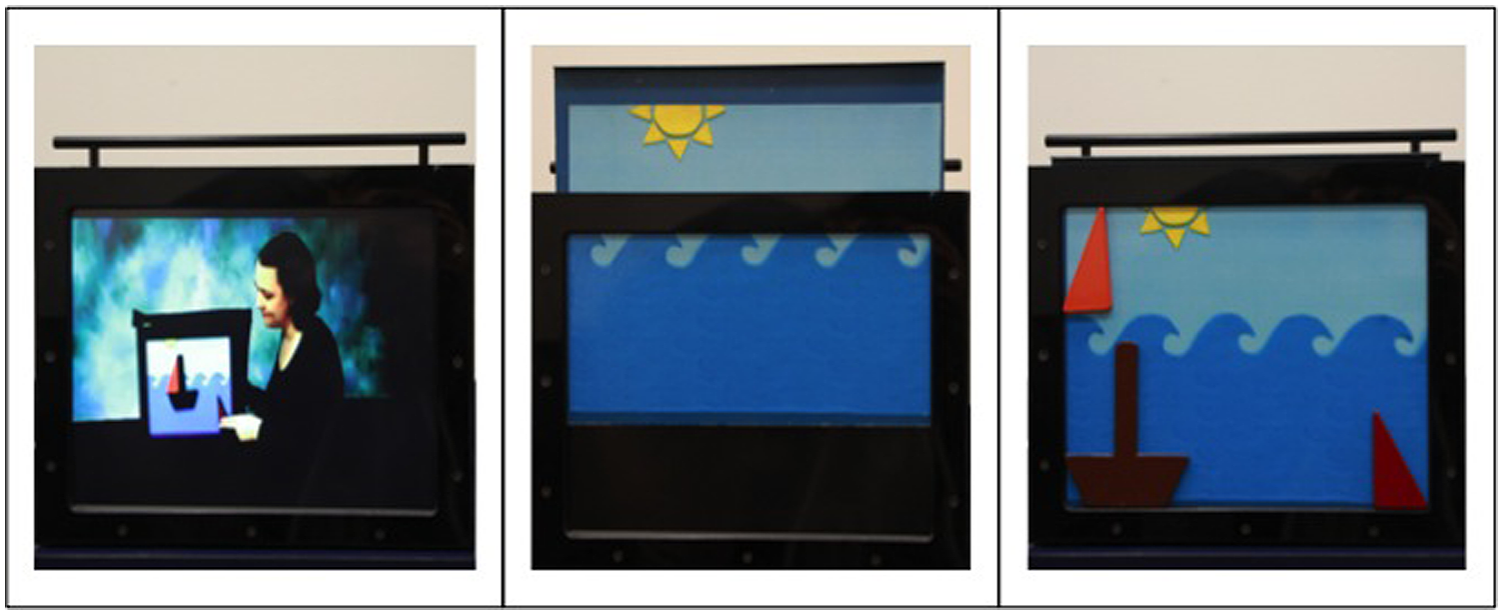
**The image on the far left depicts the apparatus with the video image displayed.** The center image depicts the apparatus with both the magnet board and video screen. The image on the far right depicts the magnet board with the boat puzzle pieces affixed to magnet board.

**FIGURE 2:**
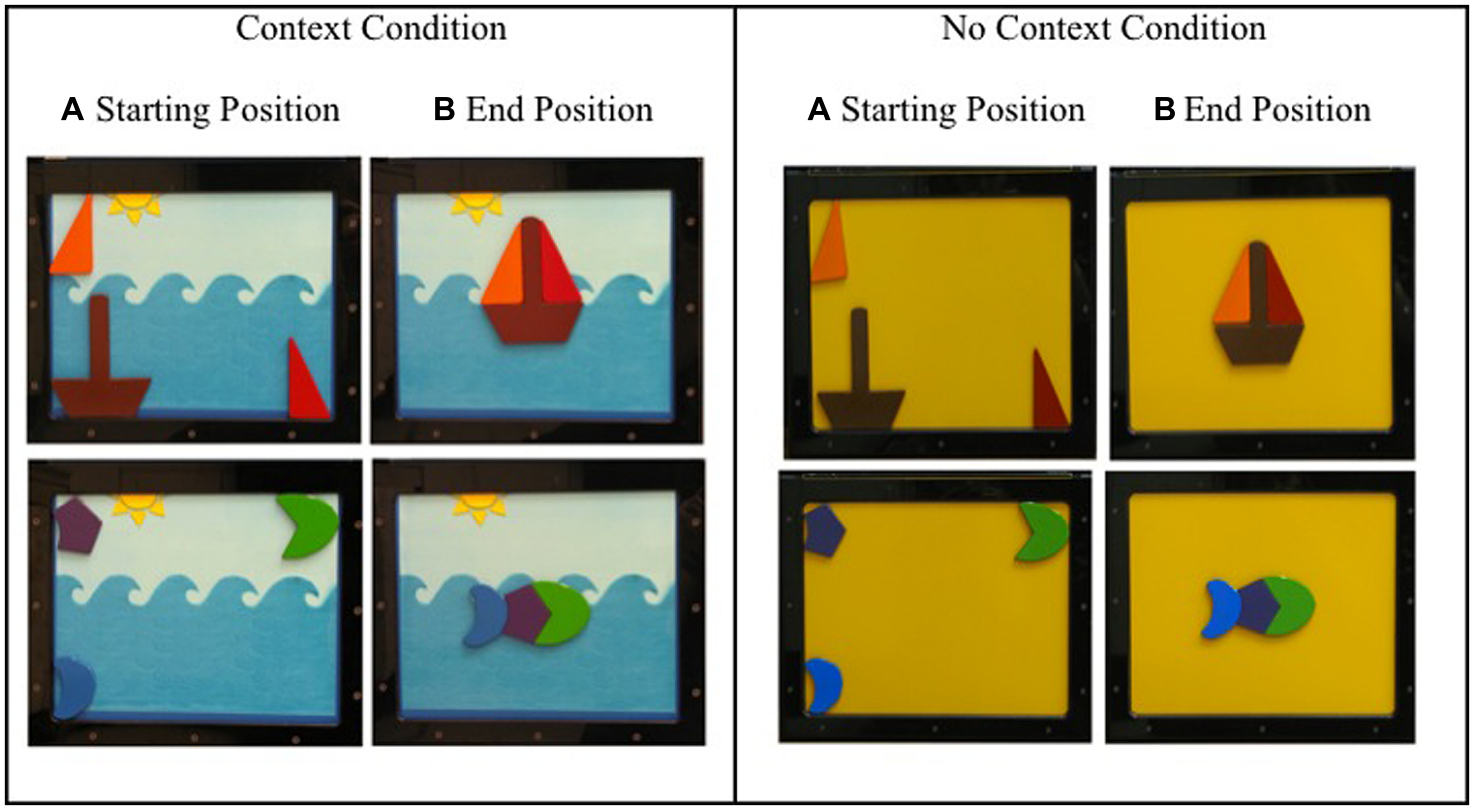
**(Left)** Context condition. The cluster of four images on the left shows the stimuli with the ocean background. **(Right)** No context condition. The cluster of four images on the right shows the stimuli with the schoolbus-yellow background. Within each context, **(A)** shows the starting position of the stimuli for the boat at the top and fish at the bottom and **(B)** shows the end position for each puzzle.

This study used a metal board inserted into a rectangular black case. The case was 35 cm tall, 42 cm wide, and 23 cm deep. The metal board could be easily slid in and out of the black case. The black case behind the metal board contained an LCD monitor that was only visible when the metal board was not in place (see **Figure 1**). The metal board was either completely school bus yellow or displayed a cartoon of the ocean. The caricature of the ocean had a light blue sky, with dark blue waves representing the ocean, and a yellow sun located at the center left of the sky. The sun was composed of one semi-circle and three triangles (see **Figure 2**).

### Stimuli

The stimuli consisted of three magnet pieces that were various shapes and colors but were the same thickness (0.5 cm). These magnets were strong enough that they stuck to the metal board, but they were weak enough so that they could be easily moved around. The pieces, when moved and connected correctly, formed either a “boat” or a “fish.” At the beginning of the trial, each piece was placed in a different corner of the metal board. For each puzzle, there were two predetermined placements for the pieces.

#### Boat

The boat puzzle consisted of three pieces: one red right triangle piece and one orange right triangle piece that represented the sails of the sailboat, and one trapezoid piece with a long thin rectangle attached at the center that represented the hull and the mask of the sail boat (see **Figure 2**).

#### Fish

The fish puzzle consisted of three pieces: one green moon shaped piece that represented the head of the fish, one purple pentagon piece that represented the body of the fish, and one blue moon shaped piece that represented the tail of the fish (see **Figure 2**).

### Vocabulary and Demographics Information

The caregiver was asked to complete a general information questionnaire (assessing SES, parental education, childcare, and language) as well as the MacArthur Communicative Development Inventory: Words and Sentences Short Form (MCDI) to measure children’s productive vocabulary (Fenson et al., 2000).

### Design

Children were randomly assigned to independent groups in order to conduct a 3 (Condition: Live, Video, Baseline) × 2 (Context: no context or context) × 2 (Age: 2.0 or 2.5 years) between-subjects design. Stimulus type (boat or fish) was counterbalanced across participants. The pieces for each stimulus set were placed in one of two arbitrarily predetermined positions that were counterbalanced across participants. Children in the baseline group did not receive a demonstration session and thus were not exposed to the stimuli until the start of the test phase.

### Procedure

All protocols were approved by the Georgetown and Binghamton University IRBs. Testing primarily occurred in the home and a small subset (*n* = 39) was tested in the laboratory. The protocol was described to parents prior to obtaining informed consent from all parents. All of the children in the study were given a brief (5–10 min) warm up play session to ensure that they were familiar and comfortable with the experimenter. The apparatus was placed on a small table about one foot high. Before the task began, the apparatus was covered by a black cloth.

#### Live Demonstration Groups

The pieces were placed on the board behind the black cloth. The experimenter lifted the cloth and showed the toddler how to put the magnet pieces together to make the “boat” or “fish.” The experimenter slid each piece by putting two fingers on the center of the piece. Every time a piece was moved, the experimenter made non-specific, fully scripted comments (“Look at this!,” “What was that?,” and “Isn’t that fun?”) to orient the child to the demonstration. After moving the pieces into place to create the “boat” or the “fish,” the experimenter covered the apparatus with the black cloth and moved the pieces back into their original locations. The demonstration was repeated three times in total; the three demonstrations together lasted approximately 50 s. After the demonstration was finished the experimenter covered the apparatus with the black cloth again and placed the pieces back into their original locations.

#### Video Demonstration Group

For this group the apparatus had the metal board removed. The experimenter lifted the cloth to reveal a monitor and played a video of another experimenter demonstrating how to put the puzzle together with the semantically meaningful context. The experimenter in the video presented the same demonstration as the experimenter in the live demonstration condition, including the use of the same scripted language. The video lasts 60 s. After the video was finished, the experimenter inserted the metal board back into the apparatus case, put the black cloth in front of the board, and placed the pieces on the board.

#### Test Phase

The test phase was the same for the video, live, and baseline groups. A short delay occurred between the end of the demonstration and the start of test. The transition was slightly longer in the transfer condition from video to magnet board (*M* = 23.12 s) than the no transfer condition resetting the pieces on the magnet (*M* = 6.81 s). The experimenter then lifted the black cloth up away from the apparatus and told the child “Now it’s your turn!” The test lasted 60 s from the first time the child touched the magnet board or any of the magnet pieces. Following the 60 s test period, the experimenter conducted a manipulation check (demonstrated the target actions one time) and then gave the child the opportunity to reproduce them. The purpose of the manipulation check was to confirm that children were capable of sliding the puzzle pieces. As part of the manipulation check, experimenters asked the child, “What did you make?” (see labeling section) to assess whether children could identify the final puzzle state as a boat or fish. The purpose of the baseline was to assess whether children spontaneously produced the target gestures or goal of connecting the puzzle pieces when they are presented with the stimuli without a demonstration.

## Results

### Coding

Imitation is operationally defined as duplicating the demonstrated actions at a rate significantly above baseline.

#### On-Task Behaviors

Each contact with a puzzle piece (beginning when a piece was touched and ending when the touch ended) was coded. Each contact was coded along two dimensions: gesture and goal. On-task behaviors excluded exploratory play (interactions where the piece was removed from the board for more than 3 s) and micro-gestures (a piece was ‘nudged,’ meaning that it was moved less than 1/6 of the board) that did not result in any type of connection.

#### Gesture Coding

Coded actions included the following categories of gestures: *correct slide*, *incorrect slide*, *strategy switch*, and *pick up and move*.

#### Goal Coding

Coded actions that connected puzzle pieces included the following categories of goals: *correct connection, target error connection,* and *connect other*. Based on 30% of all test sessions rescored by a second coder, inter-rater reliability was very good (kappas on each of the subscales; κ_gesture_ = 0.76, κ_goal_ = 0.81).

The coded goals and gestures were used to compute four dependent measures (gesture imitation, action fidelity, goal imitation, and goal efficiency). Analyses of gesture imitation and action fidelity (both derived from gesture-coded actions) are presented first followed by analyses of goal imitation and goal efficiency (both derived from goal-directed actions). The coding of action fidelity and goal efficiency is included to more precisely characterize the participants’ overall behavior during the test phase. Additionally, labeling performance and a vocabulary measure based on parental report (MCDI) was coded. **Table 1** shows the mean proportion score for each dependent measure for each condition and age group.

**Table 1:** Mean proportions and SEs for each dependent measure across age, transfer type, and context.

Measures									
		**Gesture proportion**		**Action fidelity**		**Goal proportion**		**Goal efficiency**	
**Transfer type**	**Context**	**2.0 years**	**2.5 years**	**2.0 years**	**2.5 years**	**2.0 years**	**2.5 years**	**2.0 years**	**2.5 years**
Baseline	None	0.06 (0.03)	0.08 (0.06)	0.07 (0.04)	0.05 (0.03)	0.00 (0.00)	0.00 (0.00)	0.00 (0.00)	0.00 (0.00)
	Ocean	0.00 (0.00)	0.14 (0.06)	0.00 (0.00)	0.11 (0.06)	0.00 (0.00)	0.04 (0.04)	0.00 (0.00)	0.03 (0.03)
Video	None	0.24 (0.11)	0.12 (0.06)	0.11 (0.06)	0.05 (0.03)	0.23 (0.12)	0.39 (0.13)	0.16 (0.10)	0.25 (0.10)
	Ocean	0.19 (0.08)	0.25 (0.09)	0.08 (0.04)	0.10 (0.05)	0.32 (0.12)	0.42 (0.15)	0.22 (0.09)	0.36 (0.13)
Live	None	0.40 (0.09)	0.28 (0.08)	0.24 (0.07)	0.24 (0.07)	0.24 (0.09)	0.81 (0.09)	0.11 (0.05)	0.42 (0.08)
	Ocean	0.45 (0.09)	0.31 (0.10)	0.36 (0.08)	0.12 (0.04)	0.61 (0.12)	0.57 (0.13)	0.25 (0.06)	0.24 (0.06)
**Measure totals**		0.23 (0.04)	0.20 (0.03)	0.15 (0.03)	0.11 (0.02)	0.23 (0.04)	0.38 (0.05)	0.12 (0.03)	0.22 (0.04)

#### Gesture Imitation Score

Following Dickerson et al. (2013), children received credit for each target puzzle piece that they correctly slid, up to a maximum of 3, during the 60 s test period. The resulting gesture imitation score was then converted to a proportion to allow for cross measure comparison. No additional points were given for multiple correct slides with the same puzzle piece.

#### Action Fidelity Score

To assess the rate at which correct slides were reproduced relative to other, less faithful actions, an action fidelity measure was calculated by taking the sum of all correct slides produced in the testing period (prior to reset following first puzzle completion) and dividing by all on-task behaviors produced (prior to reset following first puzzle completion). Higher proportions indicate more faithful reproduction of demonstrated actions; lower proportions indicate increasing numbers of non-demonstrated actions were produced during the test.

#### Goal Imitation Score

Following Dickerson et al. (2013), children received one point for each correct connection (maximum = 2). As with the gesture imitation score, the goal imitation score was then converted to a proportion (out of two). The goal imitation score is distinct from the gesture imitation and the action fidelity scores in that if a child used an incorrect gesture to correctly connect two puzzle pieces, they still received a point for the goal.

#### Goal Efficiency Score

This measure is calculated as all correct connections performed as a proportion of all on-task behaviors prior to first puzzle completion. This measure allows participants to be classified on a continuum, with higher proportions being indicative of highly efficient puzzle reproduction on one end to failure to reproduce the puzzle at all on the other end. For example, for the boat puzzle, a child might simply move the two sails to most efficiently complete the puzzle, but another child might imitate by first moving the brown mast and then the sails. Even less efficiently, another child may produce 20 on-task behaviors in the course of making the puzzle.

#### Vocabulary Measure

For the MCDI parental report questionnaire, percentile rank scores were calculated from raw scores using age and gender norms (Fenson et al., 2000). The mean percentile ranks were in the average range for 2 year olds (*M* = 42.31, SD = 30.59) and for 2.5-year-olds (*M* = 38.11, SD = 27.05).

#### Labeling

Coders recorded if the child generated the object label, either “fish” or “boat” (or synonyms of the object), and when identification first occurred, either during the demonstration, test, or post-test phase with minimal prompting (i.e., “What did you make?”). A score of 0 was given if no label was produced during any phase; a score of 1 was given if a child produced a label during any phase. Parental report from the MCDI collected prior to test indicated that the majority of children had fish (79%) and boat (85%) in their vocabulary.

### Data Analysis Plan

First we conducted a preliminary analysis on experimental groups on each of the four dependent measures (**Table 1**). Next we assessed whether performance between groups, both experimental and baseline, differ as a function of age (2.0, 2.5 years), transfer type (video, live), and context (ocean, none). Third, excluding baseline participants, we conducted a first order correlational analysis to assess which of our demographic factors, experimental conditions, labeling behavior, and vocabulary were associated with performance on the four dependent measures (see **Table 2**). Based on the pattern of results in our correlational analysis, we conducted a multivariate linear regression on the goal imitation measure (see **Table 3**).

**Table 2:** First order correlation between context, stimulus, age, gender, labeling, and the four dependent variables.

	Goal imitation	Goal efficiency	Gesture imitation	Action fidelity	Transfer	Context	Age	Gender	Stimulus	Label
Goal efficiency	0.854^∗∗^	–	–	–	–	–	–	–	–	–
Gesture imitation	0.169	0.014	–	–	–	–	–	–	–	–
Action fidelity	0.198^∗^	0.058	0.772^∗∗^	–	–	–	–	–	–	–
Transfer condition	–0.202^∗^	0.009	–0.260^∗∗^	–0.336^∗∗^	–	–	–	–	–	–
Context	0.088	0.063	0.044	–0.012	0.064	–	–	–	–	–
Age	0.202^∗^	0.201^∗^	–0.161	–0.178	0.060	0.009	–	–	–	–
Gender	0.013	0.033	–0.060	–0.070	–0.006	0.009	–0.063	–	–	–
Stimulus type	0.359^∗∗^	0.397^∗∗^	–0.127	–0.040	0.114	0.064	0.061	–0.118	–	–
Labeling	0.122	0.204^∗^	–0.004	–0.006	0.005	0.083	0.187	–0.210^∗∗^	0.315^∗∗^	–
MCDI	0.187	0.251^∗∗^	0.116	0.195^∗^	0.116	0.074	–0.108	–0.168	0.232^∗^	0.101

^∗^*p* ≤ 0.05, ^∗∗^*p* ≤ 0.01.

**Table 3:** The regression models for goal imitation and gesture imitation performance.

	Goal imitation				Gesture imitation			
	Unstandardized coefficient		Standardized coefficient		Unstandardized coefficient		Standardized coefficient	
	*B*	SE	β	*t*	*B*	SE	β	*t*
(Constant)	0.443	0.039		11.33^∗∗^	0.287	0.031		9.15^∗∗^
Transfer	–0.246	0.079	–0.264	–3.10^∗∗^	–0.167	0.064	–0.246	–2.62^∗^
Context	0.082	0.079	0.088	1.04	0.052	0.063	0.078	0.826
Age	0.203	0.080	0.219	2.54^∗^	–0.094	0.064	–0.140	–1.47
Stimulus	0.365	0.083	0.394	4.38^∗∗^	–0.068	0.067	–0.101	–1.02
Labeling	–0.055	0.086	–0.057	–0.63	0.035	0.069	0.050	0.500
Transfer × labeling	0.400	0.165	0.209	2.43^∗^	0.199	0.132	0.144	1.51
Transfer × age	–0.211	0.161	–0.113	–1.31	0.038	0.129	0.028	0.292

^∗^*p* ≤ 0.05, ^∗∗^*p* ≤ 0.01.

### Preliminary analyses

Preliminary analyses on gesture imitation and action fidelity revealed no main effects of gender, stimulus type, or latency between the demonstration and test session and only entered one interaction, which did not survive follow-up analyses. Therefore, gender, stimulus, and latency between demonstration and test will not be considered further for gesture imitation or action fidelity.

**FIGURE 3:**
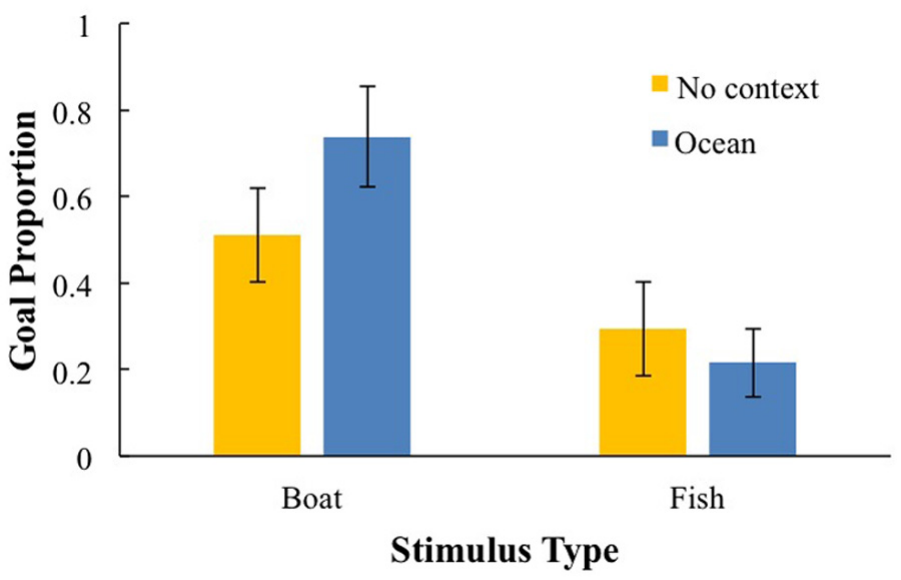
**The effect of context and stimulus type on goal proportion score**.

Preliminary analyses on goal imitation and goal efficiency, revealed no main effects of gender or latency between the demonstration and test session. These variables only entered one interaction, which did not survive follow-up analyses. For goal imitation, stimulus type did enter into significant 2-way interactions and will be analyzed further. There was a main effect of stimulus type, *F*(1,95) = 18.82, *p* < 0.001, $\eta_{p}^{2}$ = 0.17, and an interaction between context and stimulus type, *F*(1,95) = 5.90, *p* < 0.05, $\eta_{p}^{2}$ = 0.06; performance was highest for the boat puzzle in the ocean context (*M* = 0.74, SD = 0.42), which was significantly higher than the boat without context (*M* = 0.50, SD = 0.48), *p* < 0.01. Performance with the fish puzzle was not affected by context (context: *M* = 0.33, SD = 0.43; none: *M* = 0.22, SD = 0.38; see **Figure 3**). A similar pattern of results emerged for goal efficiency. These effects involving stimulus type will be discussed further; see Correlational analysis section.

### Gesture Imitation and Action Fidelity Analysis

#### Gesture Imitation

A 3 (transfer type: baseline, video, live) × 2 (age: 2.0, 2.5 years) × 2 (context: ocean, none) ANOVA on gesture imitation yielded a main effect of transfer type, *F*(2,153) = 14.45, *p* < 0.001, $\eta_{p}^{2}$ = 0.16. Gesture imitation following a ‘live’ demonstration (*M* = 0.37, SD = 0.35) was significantly higher than following a video demonstration (*M* = 0.20, SD = 0.30), which did not differ from baseline (*M* = 0.07, SD = 0.16). There was no main effect of age, *F* < 1, or context, *F* < 1, and no significant interactions.

#### Action Fidelity

A 3 (transfer type: baseline, video, live) × 2 (age: 2.0, 2.5 years) × 2 (context: ocean, none) ANOVA on action fidelity revealed a main effect of transfer type, *F*(2,153) = 13.99, *p* < 0.001, $\eta_{p}^{2}$ = 0.15. As with gesture imitation, action fidelity was higher following live (*M* = 0.24, SD = 0.27) than video demonstrations (*M* = 0.08, SD = 0.14) and baseline (*M* = 0.06, SD = 0.14). Again, the video group did not significantly exceed baseline performance, a clear demonstration of poor learning from video. Neither age nor context were significant (*F* < 1), but a 3-way interaction between age, transfer type and context was observed; *F*(2,153) = 3.74, *p* < 0.05, $\eta_{p}^{2}$ = 0.05. To follow up this 3-way interaction, we conducted Tukey HSD *post hoc* tests (*p* < 0.01). There were no differences among the baseline and video groups. Among the live demonstration groups, although it did not reach statistical significance, the effect appears to be driven by the 2-year-old context group (*M* = 0.36, SD = 0.30) that showed elevated action fidelity performance relative to the other groups: 2- and 2.5-year-olds without context (2.0: *M* = 0.24, SD = 0.32; 2.5: *M* = 0.24, SD = 0.26) and 2.5-year-olds with context (*M* = 0.12, SD = 0.14). In summary, these analyses indicate that context did not ameliorate the transfer deficit.

### Goal Imitation Score and Goal Efficiency Score Analysis

#### Goal Imitation

A 3 (transfer type: baseline, video, live) × 2 (age: 2.0, 2.5 years) × 2 (context: ocean, none) ANOVA performed on goal imitation yielded a main effect of age, *F*(1,153) = 5.96, *p* < 0.05, $\eta_{p}^{2}$ = 0.04, and transfer type, *F*(2,153) = 31.60, *p* < 0.001, $\eta_{p}^{2}$ = 0.29, but no effect of context (*F* < 1). A follow-up Tukey HSD test on transfer type demonstrated a clear transfer deficit; the live demonstration group imitated significantly more goal actions (*M* = 0.52, SD = 0.45) compared to the video group (*M* = 0.34, SD = 0.46), which was above baseline levels (*M* = 0.01, SD = 0.07). This effect was qualified by a significant three-way interaction between age, context, and condition, *F*(2,153) = 3.20, *p* < 0.05, $\eta_{p}^{2}$ = 0.04. To follow-up the 3-way interaction, we conducted Tukey HSD *post hoc* tests (*p* < 0.01). The 3-way effect was largely confined to an age-related difference in the live condition; 2-year-olds who received a context-backed demonstration (*M* = 0.61, SD = 0.45) showed higher goal performance relative to all other groups involving 2-year-olds (*M*’s ranged from 0.23–0.32). 2.5-year-olds showed the standard transfer deficit, with live conditions eliciting generally better performance than video conditions. The baseline conditions did not significantly rise above zero. Importantly, the addition of context did not ameliorate the transfer deficit at either age.

#### Goal Efficiency

A 3 (transfer type: baseline, video, live) × 2 (age: 2.0, 2.5 years) × 2 (context: ocean, none) ANOVA on goal imitation produced a main effect of age, *F*(1,153) = 5.28, *p* < 0.05, $\eta_{p}^{2}$ = 0.03 and condition, *F*(2,153) = 16.55, *p* < 0.001, $\eta_{p}^{2}$ = 0.18; live (*M* = 0.24, SD = 0.26) and video (*M* = 0.25, SD = 0.37) groups did not differ from one another, but both were significantly above baseline (*M* = 0.01, SD = 0.04). No other main effects or interactions emerged.

### Correlational Analysis

In order to assess which factors were associated with imitation performance across the four dependent measures, a first-order correlation matrix was constructed. This included demographic factors, experimental factors, and naming and vocabulary variables. Review of the correlation matrix reveals that gesture imitation and action fidelity, not surprisingly, are associated with one another, *r*(109) = 0.77, *p* < 0.01, but are associated with few of the other variables except for transfer. Goal imitation and goal efficiency, as expected, are also associated with one another, *r*(109) = 0.85, *p* < 0.01. These two are also associated with action fidelity, age, transfer, and stimulus type. This pattern of results suggests that factors predicting goal imitation may differ from those predicting gesture imitations. To explore this idea further, a regression model with goal imitation as the outcome variable was constructed. A second model was constructed with gesture imitation using the same predictors as well, to enable examination of these measures separately.

#### Predicting Goal Imitation

This analysis was conducted to identify factors associated with enhanced transfer performance. Transfer type (live, video), context (none or ocean), stimulus type (fish or boat), age (2, 2.5 years) and labeling (yes or no) were included in a multivariate linear regression on goal imitation performance. A labeling × transfer interaction term and age × transfer interaction term were entered simultaneously as well. All predictor means were centered. Interaction terms were calculated using the centered means. Although a number of first order correlations were significant (see **Table 2**), there was no multi-collinearity in the model; VIFs range from 1.03 to 1.16. Given that our prior ANOVA analyses had previously determined that neither context nor stimulus entered into significant interactions with transfer, these interaction terms were not included in the final regression model. Results from the regression are presented in **Table 3**. The overall model was significant, *F*(7,101) = 5.67, *p* < 0.001, *R* = 0.53, *R*^2^ = 0.28. As expected, transfer type (live, video) was a significant predictor, indicating the transfer deficit. There was a main effect of age; older children showed higher goal imitation overall, and of stimulus type (fish, boat), demonstrating that children connected more pieces with the boat puzzle than the fish puzzle, an effect that has been reported in prior work.

**FIGURE 4:**
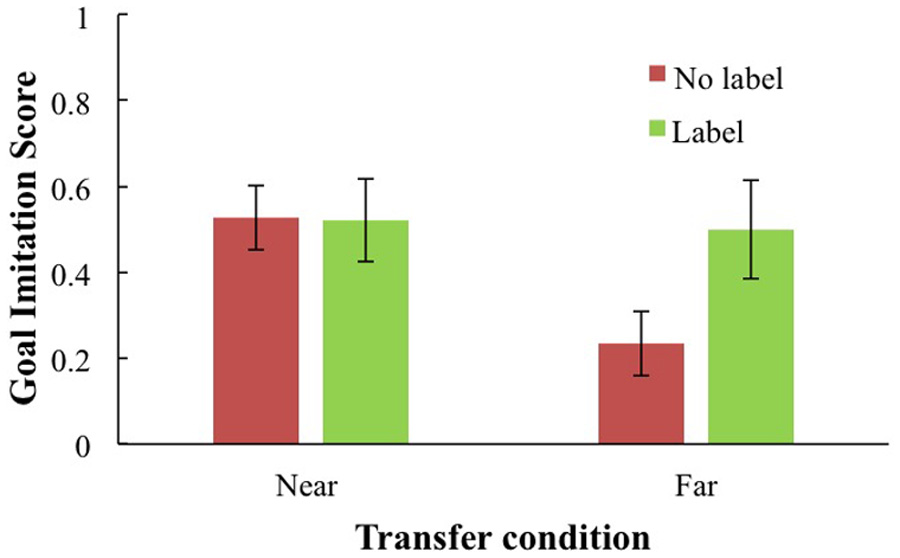
**Goal imitation proportion as a function of transfer condition (near live or far video condition) and production of self-generated label**.

Labeling alone did not predict goal imitation. There was, however, a significant interaction between transfer type and labeling: The advantage of a self-generated label was 0.40 points greater when it was combined with the transfer condition than when it was not. Follow-up regressions were conducted to examine the simple slopes for toddlers who had labeled and those who had not, as a function of transfer. Although there was a significantly negative effect when children did not self-generate a label, *F*(1,66) = 7.53, *p* < 0.04, *B* = –0.29, β = –0.32, there was no difference in the slope when children did generate a label, *F*(1,42) < 1. This analysis supports the interpretation that the *ability to label enabled these children to make the far transfer “jump.”* Thus, the impact of the transfer deficit was ameliorated for children who generated an object label during the test phase. The transfer type by self-generated label interaction is depicted in **Figure 4**. As shown, children in the far transfer (video) condition who generated a label for the puzzle produced significantly higher imitation scores than children in the video condition who did not (see **Figure 4**). No other effects were significant.

The same model was marginally significant for gesture imitation, *F*(7,101) = 2.10, *p* = 0.051, *R* = 0.36, *R*^2^ = 0.13. As shown in **Table 3**, transfer type was the only significant predictor, once again demonstrating that performance of the video group was significantly worse than the live group. No other associations were significant, including interaction terms. Comparison of the models suggests that factors that reduce the transfer deficit for goal imitation are not the same as for gesture imitation. Other models including additional interaction terms were conducted, but these interactions were not significant and the overall model did not explain more of the variance.

## General Discussion

Consistent with previous findings, this study showed that young children displayed a significant transfer deficit. Two- and 2.5-year-old children who received a video demonstration reproduced significantly fewer gestures and goals than children receiving a live demonstration. Consistent with our hypothesis, we did find an age-related effect of context in the live condition. Contrary to our hypothesis, we found that the addition of a semantically meaningful visual context did not ameliorate the transfer deficit. Importantly though, the context did not interfere with learning either. This finding is consistent with a point in development where children may form representations that contain primarily central cue information, resulting in neither a disruptive nor a facilitative effect of background context. There were individual differences in self-generation of a label that were associated with better performance for the transfer group on the puzzle task.

Rather than being impacted by context, the transfer deficit was ameliorated when children were able to generate a verbal label for the puzzle. There was a significant positive correlation between a child’s ability to generate a label for the completed puzzle and their ability to correctly connect the puzzle pieces (goal proportion). This labeling effect only facilitated performance for children in the video group, however. That is, it was the self-generated labeling of the object, and not the semantically relevant context that facilitated transfer of goal learning, highlighting the importance of a pre-existing object representation that facilitated transfer across 2D and 3D demonstration and test phases. This finding adds to the growing body of research suggesting that self-generating the object label enhances young children’s performance (e.g., Miller and Marcovitch, 2011). Other studies have also demonstrated that vocabulary size predicts object recognition, such as in Smith (2003), who reported a positive correlation between language and recognition. Smith found that although 18- to 24-month-olds (with smaller vocabularies) and children (with larger vocabularies) were able to recognize richly detailed instances of an object equally well, children with smaller *noun* vocabularies performed at chance levels when presented with a more perceptually challenging recognition task that included less iconic images of shapes. Simcock and Hayne (2002) used a ‘magic shrinking machine’ task to assess children’s understanding of the actions required to operate the box and objects that were made smaller, following a long delay. Their results, that children’s verbal reports following the delay matched their verbal skill during the encoding event rather than their verbal skill when tested 6 months or one year later (Simcock and Hayne, 2002), highlight the importance of children’s productive vocabulary at the time of encoding.

Taken together, prior research suggests that object labels may help establish abstract and dual representations of objects, as well as direct attention to relevant task details (Miller and Marcovitch, 2011). This research is consistent with our finding that the label serves as an effective retrieval cue for children in the present study on a far transfer task. Language can enhance recognition and learning under perceptually impoverished conditions and high cognitive load. Transfer distance increases cognitive load and the label acts as a cue that facilitates both encoding and retrieval (Simcock and Hayne, 2002; Hayne and Herbert, 2004; Troseth, 2010; Miller and Marcovitch, 2011).

Corresponding research on experimenter-generated verbal cues suggests that these cues are not as robust under challenging learning conditions. Bates et al. (1989) found support for the argument that congruent experimenter-generated language cues facilitated imitation performance in 1-year-olds (see also Gerson and Woodward, 2013). These results suggest that the use of relevant language enhances object recognition and imitation under conditions where there is no transfer. The same was not necessarily true, however, for a transfer task. Zack et al. (2013) found that neither a nonsense nor a meaningful object label facilitated 15-month-olds’ imitation on a touchscreen transfer task (2D to 3D or 3D to 2D). There are, however, age-related differences in the effectiveness of verbal cues. Studies of narration effects are important to consider as verbal cues have semantic or referential meaning and may be more effective retrieval cues than non-verbal auditory cues. Studies of the effect of narrative cues during an imitation task with 18- and 24-month-olds suggest infants can imitate from TV or books when verbal descriptions are not available. Additionally they can rely on verbal cues when images of the objects are absent (Simcock et al., 2011). There is likely to be a bidirectional effect whereby language affects learning and vice versa. Language development is also associated with domain-general processes such as individual differences in working memory and long-term retention. Understanding how these factors are related to transfer learning requires further empirical investigation.

The label may not be the only factor that facilitates transfer. The presence of the label suggests that the child possesses a representation of the object; not just as a single encoded exemplar, but rather, what Rosch et al. (1976) first called an *entry level category*. This indicates that the child possesses a generalizable representation of “boat” or “fish” in the present test, potentially allowing these children to access this category from either the 2D image or the 3D puzzle, in agreement with Hayne’s (2004) concept of memory flexibility. In other words, the children do not have to recognize the two instantiations as the same thing precisely, but only as exemplars of the same category. The stimulus effects described above (the boat puzzle, in general, invoked better performance than the fish puzzle, and ameliorated the transfer deficit when the child could produce the label) supports the argument that construction of the puzzle, independent of the other manipulations, affected access to a representation. The boat puzzle, with its clearly parsed sails and recognizable mast, displays a set of parts that map onto a mid-level visual representation of the type described by Biederman (1987; Hummel and Stankiewicz, 1996; see also Schacter et al., 1990; Biederman and Cooper, 1991; Biederman and Gerhardstein, 1993). The puzzle pieces that make up the fish, however, do not clearly correspond to certain recognizable parts of a fish (head or fins). Further, the pieces are of different colors, which are highly unlikely to correspond to any prior ‘fish’ exemplars that the majority of the children tested would have experienced, making access to a category more difficult even in cases where a child does possess such a category. This interpretation is bolstered by the finding that the presentation of context facilitated performance when the boat, but not the fish puzzle, was demonstrated.

In the present study, individual differences in the self-generated labels were associated with transfer performance. This outcome provides a potential explanation for how the semantic context facilitated performance on the puzzle task. This interpretation has limitations. It is possible that children who did not generate the label spontaneously may have known the label but did not express it, or that they may still benefit from a label or verbal cue being provided by the experimenter during the demonstration. Future studies could address this systematically by including labels during the demonstration phase. Also, future studies should seek to investigate whether other individual differences such as working memory or experience with puzzles are associated with performance.

Experimenter-generated nonverbal cues (i.e., visual context) in the present study did not reduce the transfer deficit but did improve overall goal performance. The lack of a main effect of nonverbal semantic context was surprising, but is consistent with similar difficulty in utilization of experimenter-generated verbal cues as discussed above and with the (non-iconic) perceptual properties of the “ocean” context used in the present study. Alternatively, this lack of a semantic context effect could be explained by accounts of developmental changes in memory binding. Research on memory binding suggests that after infancy, central and peripheral details are no longer fused, and children may disregard peripheral and contextual information and focus on more central details. A more salient foreground object may prevent toddlers from utilizing the background cues available because these cues are less salient. Consequently, children may not automatically bind the context to the memory as they did earlier in development. The attention system may focus on central details with overall less binding, resulting in neither a facilitative nor a disruptive effect of context. Processing of central and peripheral details and binding may become more flexible with further development (e.g., Sluzenski et al., 2006; Lloyd et al., 2009; Bornstein et al., 2011; Olson and Newcombe, 2014). This progression would ultimately result in a facilitatory effect of context without disruptive effects under conditions of context change. This progression may track developmental changes in the hippocampus (Olson and Newcombe, 2014; see also Chalfonte and Johnson, 1996; Mitchell et al., 2000 for discussion of age-related decline in hippocampal functioning and flexible memory binding). The developmental trajectory of memory binding during early childhood requires additional empirical attention. Additional research is also necessary to ascertain whether older children use contextual information to form flexible adult-like memories that contain both background and central information that can be used under similar complex transfer learning conditions (see also Olson and Newcombe, 2014).

Other factors more proximal to the puzzle imitation task may have limited children’s ability to utilize the contextual cues. Puzzle complexity in this task was high. It is important to note that to make our task ecologically valid we deliberately used cartoon-like and abstract representations for both the puzzle pieces and the background context. Many educational applications include animated (low iconicity) images because these images are easier to program. However, the lower iconicity of the context in the present task may have limited children’s ability to utilize contextual cues. Future studies could include more iconic representations of both stimuli (boats and fish) and context (e.g., fins and eyes on the fish or photographic images of the ocean). There are also likely to be individual differences in attention to pieces, gestures, and the context background; assessing visual attention to the context and puzzle pieces using eye-tracking may prove fruitful in this regard. The present study adds to a growing body of literature showing that the transfer deficit persists into toddlerhood (Dickerson et al., 2013, see also McGuigan et al., 2007; Moser et al., 2015). The ocean context facilitated completion of the boat puzzle relative to the fish puzzle. In addition, self-generated labeling of the puzzle (boat or fish) elevated goal performance of those in the video demonstration condition. This suggests that object identification can ameliorate the transfer deficit during toddlerhood. Understanding the nature of visuo-spatial integration (Bremner, 1978; Lockman, 2000; Kirkorian and Pempek, 2013) and spatial development more generally in early childhood has important implications for both parents and educators (see Levine et al., 2012 for related discussion). This puzzle imitation far transfer task provides a unique opportunity to examine the role of multiple factors that influence cognitive development.

## Conflict of Interest Statement

The authors declare that the research was conducted in the absence of any commercial or financial relationships that could be construed as a potential conflict of interest.

## References

[B1] Barnat S. B. Klein P. J. Meltzoff A. N. (1996). Deferred imitation across changes in context and object: memory and generalization in 14-month-old infants. *Infant Behav. Dev.* 19 241–251 10.1016/S0163-6383(96)90023-5 25147417 PMC4137786

[B2] Barnett S. M. Ceci S. J. (2002). When and where do we apply what we learn? A taxonomy for far transfer. *Psychol. Bull.* 128 612–637 10.1037/0033-2909.128.4.612 12081085

[B3] Barr R. (2010). Transfer of learning between 2D and 3D sources during infancy: informing theory and practice. *Dev. Rev.* 30 128–154 10.1016/j.dr.2010.03.001 20563302 PMC2885850

[B4] Barr R. (2013). Memory constraints on infant learning from picture books, television, and touchscreens. *Child Dev. Perspect.* 7 205–210 10.1111/cdep.12041

[B5] Barr R. Hayne H. (1999). Developmental changes in imitation from television during infancy. *Child Dev.* 70 1067–1081 10.1111/1467-8624.00079 10546335

[B6] Barr R. Muentener P. Garcia A. Fujimoto M. Chavez V. (2007). Age-related changes in deferred imitation from television by 6- to 18-month-olds. *Dev. Sci.* 10 910–921 10.1111/j.1467-7687.2007.00641.x 17973804

[B7] Bates E. Thal D. Whitesell K. Fenson L. Oakes L. (1989). Integrating language and gesture in infancy. *Dev. Psychol.* 25 1004–1019 10.1037/0012-1649.25.6.1004

[B8] Biederman I. (1972). Perceiving real-world scenes. *Science* 177 77–80 10.1126/science.177.4043.77 5041781

[B9] Biederman I. (1987). Recognition-by-components: a theory of human image understanding. *Psychol. Rev.* 94 115–147 10.1037/0033-295X.94.2.115 3575582

[B10] Biederman I. Cooper E. E. (1991). Priming contour-deleted images: evidence for intermediate representations in visual object priming. *Cogn. Psychol.* 23 393–419 10.1016/0010-0285(91)90014-F 1884597

[B11] Biederman I. Gerhardstein P. (1993). Recognizing depth-rotated objects: evidence and conditions for three-dimensional viewpoint invariance. *J. Exp. Psychol. Hum. Percept. Perform.* 19 1162–1182 10.1037/0096-1523.19.6.1162 8294886

[B12] Boller K. Rovee-Collier C. Gulya M. Prete K. (1996). Infants’ memory for context: timing effects of postevent information. *J. Exp. Child Psychol.* 63 583–602 10.1006/jecp.1996.0063 8953226

[B13] Bornstein M. H. Mash C. Arterberry M. E. (2011). Perception of object–context relations: eye-movement analyses in infants and adults. *Dev. Psychol.* 47 364 10.1037/a0021059 PMC3412542 21244146

[B14] Borovsky D. Rovee-Collier C. (1990). Contextual constraints on memory retrieval at 6 months. *Child Dev.* 61 1569–1583 10.2307/1130765 2245747

[B15] Bouton M. E. (1993). Context, time, and memory retrieval in the interference paradigms of Pavlovian learning. *Psychol. Bull.* 114 80–99 10.1037/0033-2909.114.1.80 8346330

[B16] Bremner J. G. (1978). Egocentric versus allocentric spatial coding in nine-month-old infants: factors influencing the choice of code. *Dev. Psychol.* 14 346 10.1037/0012-1649.14.4.346

[B17] Carver L. J. Meltzoff A. N. Dawson G. (2006). Event-related potential (ERP) indices of infants’ recognition of familiar and unfamiliar objects in two and three dimensions. *Dev. Sci.* 9 51–62 10.1111/j.1467-7687.2005.00463.x 16445396 PMC1475557

[B18] Chalfonte B. L. Johnson M. K. (1996). Feature memory and binding in young and older adults. *Mem. Cognit.* 24 403–416 10.3758/BF03200930 8757490

[B19] Chun M. M. Jiang Y. (1998). Contextual cueing: implicit learning and memory of visual context guides spatial attention. *Cogn. Psychol.* 36 28–71 10.1006/cogp.1998.0681 9679076

[B20] Cohen N. J. Eichenbaum H. (1993). *Memory, Amnesia, and the Hippocampal System*. Cambridge, MA: MIT press.

[B21] Csibra G. Gergely G. (2006). “Social learning and social cognition: the case for pedagogy,” in *Processes of Change in Brain and Cognitive Development. Attention and Performance XXI* eds Munakata Y. Johnson M. H. (Oxford: Oxford University Press) 249–274.

[B22] Deloache J. Simcock G. Marzolf D. P. (2004). Transfer by very young children in the symbolic retrieval task. *Child Dev.* 75 1708–1718 10.1111/j.1467-8624.2004.00811.x 15566374

[B23] Dickerson K. Gerhardstein P. Zack E. Barr R. (2013). Age-related changes in learning across early childhood: a new imitation task. *Dev. Psychobiol.* 55 719–732 10.1002/dev.21068 22786801

[B24] Fenson L. Bates E. Dale P. Goodman J. Reznick J. S. Thal D. (2000). Reply: measuring variability in early child language: don’t shoot the messenger. *Child Dev.* 71 323–328 10.1111/1467-8624.00147 10834467

[B25] Flynn E. Whiten A. (2008). Imitation of hierarchical structure versus component details of complex actions by 3- and 5-year-olds. *J. Exp. Child Psychol.* 101 228–240 10.1016/j.jecp.2008.05.009 18639887

[B26] Gerson S. A. Woodward A. L. (2013). The goal trumps the means: highlighting goals is more beneficial than highlighting means in means-end training. *Infancy* 18 289–302 10.1111/j.1532-7078.2012.00112.x 23723734 PMC3665361

[B27] Hartshorn K. Rovee-Collier C. (1997). Infant learning and long-term memory at 6 months: a confirming analysis. *Dev. Psychobiol.* 30 71–85 10.1002/(SICI)1098-2302(199701)30:1<71::AID-DEV7>3.0.CO;2-S 8989534

[B28] Hayne H. (2004). Infant memory development: implications for childhood amnesia. *Dev. Rev.* 24 33–73 10.1016/j.dr.2003.09.007

[B29] Hayne H. Boniface J. Barr R. (2000). The development of declarative memory in human infants: age-related changes in deffered imitation. *Behav. Neurosci.* 114 77–83 10.1037/0735-7044.114.1.77 10718263

[B30] Hayne H. Herbert J. (2004). Verbal cues facilitate memory retrieval during infancy. *J. Exp. Child Psychol.* 89 127–139 10.1016/j.jecp.2004.06.002 15388302

[B31] Hummel J. E. Stankiewicz B. J. (1996). “An architecture for rapid, hierarchical structural description,” in *Attention and Performance XVI: Information Integration in Perception and Communication* eds Inui T. McClelland J. L. (Cambridge, MA: MIT Press) 93–121.

[B32] Kirkorian H. L. Pempek T. A. (2013). Toddlers and touch screens: potential for early learning? *Zero three* 33 32–37.

[B33] Learmonth A. E. Lamberth R. Rovee Collier C. (2004). Generalization of deferred imitation during the first year of life. *J. Exp. Child Psychol.* 88 297–318 10.1016/j.jecp.2004.04.004 15265678

[B34] Levine S. C. Ratliff K. R. Huttenlocher J. Cannon J. (2012). Early puzzle play: a predictor of preschoolers’ spatial transformation skill. *Dev. Psychol.* 48 530–542 10.1037/a0025913 22040312 PMC3289766

[B35] Lloyd M. E. Doydum A. O. Newcombe N. S. (2009). Memory binding in early childhood: evidence for a retrieval deficit. *Child Dev.* 80 1321–1328 10.1111/j.1467-8624.2009.01353.x 19765002

[B36] Lockman J. J. (2000). A perception–action perspective on tool use development. *Child Dev.* 71 137–144 10.1111/1467-8624.00127 10836567

[B37] McGuigan N. Whiten A. Flynn E. Horner V. (2007). Imitation of causally opaque versus causally transparent tool use by 3- and 5-year-old children. *Cogn. Dev.* 22 353–364 10.1016/j.cogdev.2007.01.001

[B38] Miller S. E. Marcovitch S. (2011). Toddlers benefit from labeling on an executive function search task. *J. Exp. Child Psychol.* 108 580–592 10.1016/j.jecp.2010.10.008 21112597 PMC3042530

[B39] Mitchell K. J. Johnson M. K. Raye C. L. Mather M. D’Esposito M. (2000). Aging and reflective processes of working memory: binding and test load deficits. *Psychol. Aging* 15 527–541 10.1037/0882-7974.15.3.527 11014715

[B40] Moser A. Zimmermann L. Dickerson K. Grenell A. Barr R. Gerhardstein P. (2015). They can interact, but can they learn? Toddlers’ transfer learning from touchscreens and television. *J. Exp. Child Psychol.* 10.1016/j.jecp.2015.04.002 25978678

[B41] Nakao K. Treas J. (1994). Updating occupational prestige and socioeconomic scores: how the new measures measure up. *Sociol. Methodol.* 24 1–72.

[B42] Newcombe N. S. Balcomb F. Ferrara K. Hansen M. Koski J. (2014). Two rooms, two representations? Episodic-like memory in toddlers and preschoolers. *Dev. Sci.* 17 743–756 10.1111/desc.12162 24628962

[B43] Nielsen M. Simcock G. Jenkins L. (2008). The effect of social engagement on 24-month-olds’ imitation from live and televised models. *Dev. Sci.* 11 722–731 10.1111/j.1467-7687.2008.00722.x 18801128

[B44] Oliva A. Torralba A. (2007). The role of context in object recognition. *Trends Cogn. Sci.* 11 520–537 10.1016/j.tics.2007.09.009 18024143

[B45] Olson I. R. Newcombe N. S. (2014). “Binding together the elements of episodes: relational memory and the developmental trajectory of the hippocampus,” in *The Wiley Handbook on the Development of Children’s Memory* eds Bauer P. J. Fivush R. (Chichester: Wiley-Blackwell) 285–308 10.1002/9781118597705.ch13

[B46] Pereira A. F. Smith L. B. (2009). Developmental changes in visual object recognition between 18 and 24 months of age. *Dev. Sci.* 12 67–80 10.1111/j.1467-7687.2008.00747.x 19120414 PMC2888029

[B47] Raj V. Bell M. A. (2010). Cognitive processes supporting episodic memory formation in childhood: the role of source memory, binding, and executive functioning. *Dev. Rev.* 30 384–402 10.1016/j.dr.2011.02.001

[B48] Rescorla R. A. Wagner A. W. (1972). “A theory of Pavlovian conditioning: variations in the effectiveness of reinforcement and nonreinforcement,” in *Classical Conditioning II: Current Research and Theory* eds Black A. H. Prokasy W. F. (New York, NY: Appleton-Century-Crofts) 64–99.

[B49] Richmond J. Nelson C. A. (2009). Relational memory during infancy: evidence from eye tracking. *Dev. Sci.* 12 549–556 10.1111/j.1467-7687.2009.00795.x 19635082

[B50] Rideout V. (2013). *Zero to Eight: Children’s Media Use in America 2013*. San Francisco, CA: Common Sense Media.

[B51] Rideout V. (2014). *Learning at Home: Families’ Educational Media Use in America*. New York, NY: Joan Ganz Cooney Center.

[B52] Rosch E. Mervis C. B. Gray W. Johnson D. Boyes-Braem P. (1976). Basic objects in natural categories. *Cogn. Psychol.* 8 382–439 10.1016/0010-0285(76)90013-X

[B53] Schacter D. L. Cooper L. A. Delaney S. M. (1990). Implicit memory for visual objects and the structural description system. *Bull. Psychon. Soc.* 28 367–372 10.3758/BF03334043

[B54] Shields P. J. Rovee-Collier C. (1992). Long-term memory for context-specific category information at 6 months. *Child Dev.* 63 245–259 10.2307/1131476 1611931

[B55] Simcock G. DeLoache J. (2006). Get the picture? The effects of iconicity on toddlers’ reenactment from picture books. *Dev. Psychol.* 42 1352–1357 10.1037/0012-1649.42.6.1352 17087568

[B56] Simcock G. Dooley M. (2007). Generalization of learning from picture books to novel test conditions by 18-and 24-month-old children. *Dev. Psychol.* 43 1568–1578 10.1037/0012-1649.43.6.1568 18020833

[B57] Simcock G. Garrity K. Barr R. (2011). The effect of narrative cues on infants’ imitation from television and picture books. *Child Dev.* 82 1607–1619 10.1111/j.1467-8624.2011.01636.x 21883157 PMC3170082

[B58] Simcock G. Hayne H. (2002). Breaking the barrier? Children fail to translate their preverbal memories into language. *Psychol. Sci.* 13 225–231 10.1111/1467-9280.00442 12009042

[B59] Sluzenski J. Newcombe N. S. Kovacs S. L. (2006). Binding, relational memory, and recall of naturalistic events: a developmental perspective. *J. Exp. Psychol. Learn. Mem. Cogn.* 32 89–100 10.1037/0278-7393.32.1.89 16478343

[B60] Sluzenski J. Newcombe N. S. Satlow E. (2004). Knowing where things are in the second year of life: implications for hippocampal development. *J. Cogn. Neurosci.* 16 1443–1451 10.1162/0898929042304804 15509389

[B61] Smith L. B. (2003). Learning to recognize objects. *Psychol. Sci.* 14 244–250 10.1111/1467-9280.03439 12741748

[B62] Spear N. E. McKinzie D. L. (1994). “Intersensory integration in the infant rat,” in *The Development of Intersensory Perception: Comparative Perspectives* eds Lewkowicz D. J. Lickliter R. (Hillsdale, NJ: Erlbaum) 133–161.

[B63] Subiaul F. Anderson S. Brandt J. Elkins J. (2012). Multiple imitation mechanisms in children. *Dev. Psychol.* 48 1165–1179 10.1037/a0026646 22201448

[B64] Suddendorf T. Simcock G. Nielsen M. (2007). Visual self-recognition in mirrors and live videos: evidence for a developmental asynchrony. *Cogn. Dev.* 22 185–196 10.1016/j.cogdev.2006.09.003

[B65] Tennie C. Call J. Tomasello M. (2006). Push or pull: imitation vs. emulation in great apes and human children. *Ethology* 112 1159–1169 10.1111/j.1439-0310.2006.01269.x

[B66] Troseth G. L. (2010). Is it life or is it Memorex? Video as a representation of reality. *Dev. Rev.* 30 155–175 10.1016/j.dr.2010.03.007

[B67] Troseth G. L. DeLoache J. S. (1998). The medium can obscure the message: young children’s understanding of video. *Child Dev.* 69 950–965 10.1111/j.1467-8624.1998.tb06153.x 9768480

[B68] Troseth G. L. Saylor M. M. Archer A. H. (2006). Young children’s use of video as a source of socially relevant information. *Child Dev.* 77 786–799 10.1111/j.1467-8624.2006.00903.x 16686801

[B69] Zack E. Barr R. Gerhardstein P. Dickerson K. Meltzoff A. N. (2009). Infant imitation from television using novel touch screen technology. *Br. J. Dev. Psychol.* 27 13–26 10.1348/026151008X334700 19972660 PMC2821208

[B70] Zack E. Gerhardstein P. Meltzoff A. N. Barr R. (2013). 15-month-olds’ transfer of learning between touch screen and real-world displays: language cues and cognitive loads. *Scand. J. Psychol.* 54 20–25 10.1111/sjop.12001 23121508 PMC3547135

